# The Interplay Among Biomolecules and Nanomaterials

**DOI:** 10.3390/ijms26168105

**Published:** 2025-08-21

**Authors:** Ran Jia

**Affiliations:** Institute of Theoretical Chemistry, College of Chemistry, Jilin University, Changchun 130023, China; jiaran@jlu.edu.cn

The unintentional application of nanomaterials by humans dates back over two millennia, though the formal concept emerged only in the late 20th century. At the nanoscale, materials exhibit pronounced quantum confinement effects, coupled with high specific surface area and abundant active sites. These unique attributes lead to physicochemical properties in nanomaterial systems that diverge significantly from their macroscopic counterparts. As a result, nanomaterials have revolutionized numerous fields, including energy, healthcare, electronics, and environmental science.

However, the intrinsic small size and unique properties of nanomaterials make them highly susceptible to interactions with biomolecules—with both beneficial and detrimental implications. Biomolecules span a vast spectrum, from larger systems like peptides, proteins, DNA, nucleic acids, and polysaccharides, to smaller entities such as amino acids, nucleotides, monosaccharides, lipid molecules, organic acids, vitamins, metabolic intermediates, signaling molecules, and neurotransmitters. On the positive side, the application of nanomaterials in medicine has been extensively explored and validated, particularly for treating challenging conditions like tumors, where conventional methods often demonstrate limited efficacy. For instance, gold nanoparticles have been successfully employed to suppress the overexpression of the CA IX isoform in hypoxic cancer cells, thereby facilitating the imaging and treatment of hypoxic tumors [1]. Furthermore, leveraging the strong near-infrared (700–1100 nm) absorption properties of single-walled carbon nanotubes (SWCNTs) allows for the selectively targeted destruction of cancer cells marked by tumor biomarkers, effectively combining drug delivery with photothermal effects [2]. Beyond therapeutic interventions, novel diagnostic approaches have emerged. Modified gold nanoparticles or two-dimensional transition metal dichalcogenides, for example, can sense characteristic organic small molecules in human breath, enabling the crucial screening of lung cancer patients without destructive injuries [3,4].

Nanomaterials possess a dual nature: promise and peril. Nanoparticles have indeed been effectively utilized for antibacterial applications. However, this very utility has simultaneously sparked extensive research and considerable concerns regarding the biotoxicity of nanomaterials [5]. The toxicity of nanomaterials and their environmental ramifications are critical issues demanding rigorous scrutiny. In chemistry and chemical engineering, nanomaterials are frequently employed as highly efficient catalysts. Consequently, when nanoparticles are dispersed in natural or living environments, they can catalyze the generation of free radicals in certain species. This can subsequently lead to oxidative stress and inflict damage upon essential biological components such as lipids, proteins, and DNA [6].

The biotoxicity of nanomaterials is intricately linked to their physicochemical properties, including size, charge, and surface functional groups. Given their exceedingly small dimensions, nanoparticles can readily be internalized by cells, potentially interacting with DNA to alter its structure and function [7]. Intriguingly, studies utilizing optical absorption spectroscopy, fluorescence spectroscopy, and atomic force microscopy have revealed that single-stranded DNA (ssDNA) effectively disperses single-walled carbon nanotubes (SWCNTs) in water. This occurs through π-stacking interactions where ssDNA binds to the carbon nanotube surface, forming a helical wrap. The resulting binding free energy is comparable to that observed between two carbon nanotubes themselves [8]. When nanomaterials enter biological media, they swiftly form a protein-rich corona, which fundamentally alters their pharmacokinetics [9,10]. The surface charge (zeta potential) of these nanoparticles plays a crucial role in determining both their stability in suspension and their in vivo toxicity [11]. Their minute size also dictates their biological fate: nanoparticles smaller than 10 nm possess the ability to traverse the blood–brain barrier, while those under 5 nm are rapidly cleared by the kidneys. While this rapid renal clearance shortens their circulation time, it also raises concerns about potential adverse effects on renal function [11].

Extensive research has explored the toxicity of carbon nanotubes (CNTs), yielding varied results contingent on the specific geometric and chemical structural properties of the CNT systems under investigation [12,13]. Similarly, graphene-based materials have been widely examined for their antibacterial capabilities. However, some studies have presented a counterintuitive finding: *Escherichia coli* can actually develop resistance to graphene oxide. This occurs because the dense biofilm formed around the material may inadvertently promote bacterial proliferation [14]. Nanotoxicity is not an inherent universal property of all nanomaterials; rather, it is a highly specific outcome resulting from the complex interplay of their unique physical and chemical attributes. This crucial distinction means that addressing toxicity concerns does not require avoiding nanomaterials altogether. Instead, it necessitates the rational design of nanomaterials with tailored physicochemical properties to minimize adverse effects and enhance biocompatibility.

To comprehensively understand the impact of nanomaterials on biological systems, it is imperative to elucidate the intricate interaction mechanisms between nanomaterials and biomolecules at the microscopic level. While experiments clearly demonstrate graphene’s efficacy in eliminating *Escherichia coli*, only through the study of underlying mechanisms can we uncover that cell membrane rupture is not caused by the physical slicing action of graphene nanosheets. Instead, phospholipid molecules within the membrane are strongly attracted by the delocalized π-electrons on graphene’s surface. This attraction leads to their detachment from the lipid bilayer and subsequent adhesion to the graphene, ultimately culminating in membrane disruption and cell death [15]. This case powerfully exemplifies the critical importance of investigating nanomaterial–biomolecule interactions beyond macroscopic observations.

Such critical research demands precise characterization of nanomaterial systems and a profound, molecular-level understanding of their interactions with biological systems. To summarize the latest advancements in applying nanomaterials for biomolecular research and to further propel progress in this vital field, we launched the Special Issue titled The Interplay Among Biomolecules and Nanomaterials.

## Data Availability

No new data were created or analyzed in this study. Data sharing is not applicable to this article.
